# Maize genomes to fields (G2F): 2014–2017 field seasons: genotype, phenotype, climatic, soil, and inbred ear image datasets

**DOI:** 10.1186/s13104-020-4922-8

**Published:** 2020-02-12

**Authors:** Bridget A. McFarland, Naser AlKhalifah, Martin Bohn, Jessica Bubert, Edward S. Buckler, Ignacio Ciampitti, Jode Edwards, David Ertl, Joseph L. Gage, Celeste M. Falcon, Sherry Flint-Garcia, Michael A. Gore, Christopher Graham, Candice N. Hirsch, James B. Holland, Elizabeth Hood, David Hooker, Diego Jarquin, Shawn M. Kaeppler, Joseph Knoll, Greg Kruger, Nick Lauter, Elizabeth C. Lee, Dayane C. Lima, Aaron Lorenz, Jonathan P. Lynch, John McKay, Nathan D. Miller, Stephen P. Moose, Seth C. Murray, Rebecca Nelson, Christina Poudyal, Torbert Rocheford, Oscar Rodriguez, Maria Cinta Romay, James C. Schnable, Patrick S. Schnable, Brian Scully, Rajandeep Sekhon, Kevin Silverstein, Maninder Singh, Margaret Smith, Edgar P. Spalding, Nathan Springer, Kurt Thelen, Peter Thomison, Mitchell Tuinstra, Jason Wallace, Ramona Walls, David Wills, Randall J. Wisser, Wenwei Xu, Cheng-Ting Yeh, Natalia de Leon

**Affiliations:** 1 University of Wisconsin, Madison, WI 53706 USA; 2 University of Illinois at Urbana-Champaign, Urbana, IL 61801 USA; 3 Cornell University, Ithaca, NY 14853 USA; 4 USDA-ARS, Beltsville, MD USA; 5 Kansas State University, Manhattan, KS 66502 USA; 6 Iowa State University, Ames, IA 50011 USA; 7 Iowa Corn Growers Association, Johnston, IA 50131 USA; 8 University of Missouri, Columbia, MO 65211 USA; 9 South Dakota State University, Rapid City, SD 57702 USA; 10 University of Minnesota, St. Paul, MN 55108 USA; 11 North Carolina State University, Raleigh, NC 27695 USA; 12 Arkansas State University, Jonesboro, AR 72401 USA; 13 University of Guelph, Ridgetown, ON Canada; 14 University of Nebraska, Lincoln, NE 68583 USA; 15 Pennsylvania State University, State College, PA 16802 USA; 16 Colorado State University, Fort Collins, CO 80523 USA; 17 Texas A&M University, College Station, TX 77843 USA; 18 Purdue University, West Lafayette, IN 47907 USA; 19 University of Florida, Gainesville, FL 32611 USA; 20 Clemson University, Clemson, SC 29634 USA; 21 Michigan State University, East Lansing, MI 48824 USA; 22 Ohio State University, Columbus, OH 43210 USA; 23 University of Georgia, Athens, GA 30602 USA; 24 University of Arizona, Tucson, AZ 85721 USA; 25 University of Delaware, Newark, DE 19716 USA

**Keywords:** Maize, Genome, Genotype, GBS, G × E, Hybrid, Inbred, Phenotype, Environment, Field metadata

## Abstract

**Objectives:**

Advanced tools and resources are needed to efficiently and sustainably produce food for an increasing world population in the context of variable environmental conditions. The maize genomes to fields (G2F) initiative is a multi-institutional initiative effort that seeks to approach this challenge by developing a flexible and distributed infrastructure addressing emerging problems. G2F has generated large-scale phenotypic, genotypic, and environmental datasets using publicly available inbred lines and hybrids evaluated through a network of collaborators that are part of the G2F’s genotype-by-environment (G × E) project. This report covers the public release of datasets for 2014–2017.

**Data description:**

Datasets include inbred genotypic information; phenotypic, climatic, and soil measurements and metadata information for each testing location across years. For a subset of inbreds in 2014 and 2015, yield component phenotypes were quantified by image analysis. Data released are accompanied by README descriptions. For genotypic and phenotypic data, both raw data and a version without outliers are reported. For climatic data, a version calibrated to the nearest airport weather station and a version without outliers are reported. The 2014 and 2015 datasets are updated versions from the previously released files [1] while 2016 and 2017 datasets are newly available to the public.

## Objective

Genomes to fields (G2F) is a multi-institutional, public collaborative to develop information and tools that support the translation of maize (*Zea mays* L.) genomic information into relevant phenotypes for the benefit of growers, consumers, and society. Building on existing maize genome sequence resources, the project focuses on developing approaches to improve phenomic predictability and facilitate the development and deployment of tools and resources that help address fundamental problems of sustainable agricultural productivity. Specific projects within G2F involve collaboration from research fields such as genetics, genomics, plant physiology, agronomy, climatology and crop modeling, computational sciences, statistics, and engineering.

As part of this effort, the G2F G × E project has collected, utilized, and shared multi-year, large-scale genotypic, phenotypic, environmental, and metadata datasets. The datasets described here were generated using standard formats between 2014 and 2017. For each of the testing locations, metadata and soil characterization are also included. During these four growing seasons, over 55,000 plots across 68 unique locations were used to evaluate inbred and hybrid plants. The resulting datasets are unique as they represent, to our knowledge, the most extensive publicly available datasets of their kind in maize, reporting a consistent set of traits across common sets of fully genotyped germplasm across many locations, along with relevant information reported down to the level of specific plots. Making these datasets publicly available is expected to enable researchers to conduct novel data analyses and develop tools using the curated and organized data described here. The 2014 and 2015 datasets are recently updated versions from previously released files (AlKhalifah et al. in BMC Res Notes 11:452, 2018) while 2016 and 2017 datasets are newly available to the public.

## Data description

Online forms were developed for logging field site coordinates, field management metadata, and other site-specific information. Datasets include:

- Genotypic information for inbreds (with and without imputation): This includes single nucleotide polymorphism (SNP) information generated using a genotyping-by-sequence (GBS) method [2] for the inbreds used to produce the hybrids tested across all locations. Data is formatted to be readily analyzed using the TASSEL software [3].
- Phenotypic measurements for inbreds and hybrids: A handbook of instructions for making traditional phenotypic measurements (reviewed in [4]) is available via the G2F website [5]. Standard traits include stand count, stalk lodging, root lodging, days to anthesis, days to silking, ear height, plant height, plot weight, grain moisture, test weight, and estimated grain yield. Datatypes reported as both raw files and files with outliers removed are described in README files. Additionally, a set of ear, cob, and kernel measurements was made using flatbed scanners and a machine vision platform to quantify components of yield [6]. These data are reported in millimeters with shape descriptors reported as principal components of contour data points. Cob color was reported as RGB (red/green/blue) pixel values. Kernel row number, counted manually, is reported as an integer.
- Environmental data: Data was collected using WatchDog 2700 weather stations (Spectrum Technologies) measuring at 30-min intervals from planting through harvest at each location. Collected information includes wind speed, direction, and gust; air temperature, dewpoint, and relative humidity; rainfall; and photoperiod. Data are reported based on calibration derived from nearby National Weather Service (NWS) Automated Surface Observing Systems (ASOS) airport weather stations and cleaned by removing obvious artifacts from the calibrated dataset.
- Soil characterizations: Information was first collected in 2015. Measurements include plow depth, pH, buffered pH, organic matter, texture and nitrogen, phosphorous, potassium, sulfur, and sodium levels (in parts per million).
- The previously released 2014 and 2015 datasets have been updated through additional quality control of the phenotypic and environmental datasets, the addition of missing site-specific field information and an update of the genotypic data to version 4 of the B73 reference genome.

The 2014–2017 datasets are publicly available via CyVerse/iPlant [7] with files and access links as shown in Table 1.

**Table 1:** Overview of data file/data set

Label	Name of data file/data set	File types (Extension)	Data repository and identifier
2014 Planting season	_readme.txt	.txt	CyVerse [8] (10.25739/9wjm-eq41)
	/a._2014_hybrid_phenotypic_data	directory	
	g2f_2014_hybrid_data_clean.csv	.csv	
	g2f_2014_hybrid_raw.csv	.csv	
	/b._2014_weather_data	directory	
	g2f_2014_weather.csv	.csv	
	/c._2014_inbred_phenotypic_data	directory	
	g2f_2014_hybrid_data_clean.csv	.csv	
	g2f_2014_hybrid_raw.csv	.csv	
	/z._2014_supplemental_info	directory	
	g2f_2014_field_characteristics.csv	.csv	
2015 Planting season	_readme.txt	.txt	CyVerse [9] (10.25739/kjsn-dz84)
	/a._2015_hybrid_phenotypic_data	directory	
	g2f_2015_hybrid_data_clean.csv	.csv	
	g2f_2015_hybrid_raw.csv	.csv	
	/b._2015_weather_data	directory	
	g2f_2015_weather.csv	.csv	
	/c._2015_inbred_phenotypic_data	directory	
	g2f_2015_hybrid_data_clean.csv	.csv	
	g2f_2015_hybrid_raw.csv	.csv	
	/d._2015_soil_data	directory	
	g2f_2016_soil_data.txt	.txt	
	g2f_2016_soil_data.csv	.csv	
	z._2015_supplemental_info	directory	
	g2f_2015_cooperator_list.csv	.csv	
	g2f_2015_field_irrigation.csv	.csv	
	g2f_2015_field_metadata.csv	.csv	
	g2f_2015_supplemental_information.txt	.csv	
2016 Planting season	_readme.txt	.txt	CyVerse [10] (10.25739/yjnh-kt21)
	/a._2016_hybrid_phenotypic_data	directory	
	g2f_2016_hybrid_data_clean.csv	.csv	
	g2f_2016_hybrid_raw.csv	.csv	
	/c._2016_weather_data	directory	
	g2f_2016_weather.csv	.csv	
	/c._2016_soil_data	directory	
	g2f_2016_soil_data.txt	.txt	
	g2f_2016_soil_data_clean.csv	.csv	
	g2f_2016_soil_data_raw.csv	.csv	
	/z._2016_supplemental_info	directory	
	g2f_2016_supplemental_information.txt	.txt	
	g2f_2016_agronomic_information.csv	.csv	
	g2f_2016_cooperators_list.csv	.csv	
	g2f_2016_field_metadata.csv	.csv	
2017 Planting season	_readme.txt	txt	CyVerse [11] (10.25739/w560-2114)
	/a._2017_hybrid_phenotypic_data	directory	
	g2f_2017_hybrid_data_clean.csv	.csv	
	g2f_2017_hybrid_data_raw.csv	.csv	
	/b._2017_weather_data	directory	
	g2f_2017_weather_data.csv	.txt	
	/c._2017_soil_data	directory	
	g2f_2017_soil_data.txt	.txt	
	g2f_2017_soil_data_clean.csv	.csv	
	g2f_2017_soil_data_raw.csv	.csv	
	/d._2017_genotypic_data	directory	
	g2f_2017_gbs_hybrid_codes.xlsx	.xlsx	
	g2f_2017_ZeaGBSv27_Imputed_ABPv4.h5	.h5	
	g2f_2017_ZeaGBSv27_Imputed_ABPv4.h5.zip	.zip	
	g2f_2017_ZeaGBSv27_Raw_ABPv4.h5	.h5	
	g2f_2017_ZeaGBSv27_Raw_ABPv4.h5.zip	.zip	
	/z._2017_supplemental_info	directory	
	g2f_2017_supplemental_information.txt	.txt	
	g2f_2017_agronomic_information.csv	.csv	
	g2f_2017_cooperators_list.csv	.csv	
	g2f_2017_field_metadata.csv	.csv	
2014 and 2015 Inbred ear imaging	_readme.txt	.txt	CyVerse [12] (10.7946/P2C34P)
	2014_2015_compiledData.tar.gz	.tar.gz	
	2014_gxe_compiledDataAndFileNames.csv	.csv	
	2014_gxe_compiledDataAndFileNames_Raw.csv	.csv	
	2015_gxe_compiledDataAndFileNames.csv	.csv	
	2015_gxe_compiledDataAndFileNames_Raw.csv	.csv	
	CEK_Data_Files.tar.gz	.tar.gz	
	/cob	directory	
	_cob.txt	txt	
	cob.tar.gz	.tar.gz	
	cob_01of05.tar.gz	.tar.gz	
	cob_02of05.tar.gz	.tar.gz	
	cob_03of05.tar.gz	.tar.gz	
	cob_04of05.tar.gz	.tar.gz	
	cob_05of05.tar.gz	.tar.gz	
	/ear	directory	
	_ear.txt	.txt	
	ear.tar.gz	tar.gz	
	ear_01of08.tar.gz	tar.gz	
	ear_02of08.tar.gz	tar.gz	
	ear_03of08.tar.gz	tar.gz	
	ear_04of08.tar.gz	tar.gz	
	ear_05of08.tar.gz	tar.gz	
	ear_06of08.tar.gz	tar.gz	
	ear_07of08.tar.gz	tar.gz	
	ear_08of08.tar.gz	tar.gz	
	/kernel	directory	
	_kernel.txt	.txt	
	kernel.tar.gz	tar.gz	
	kernel_01of05.tar.gz	tar.gz	
	kernel_02of05.tar.gz	tar.gz	
	kernel_03of05.tar.gz	tar.gz	
	kernel_04of05.tar.gz	tar.gz	
	kernel_05of05.tar.gz	tar.gz	

As the number of collaborators, plots evaluated and research questions across this project grows, it is anticipated that the variety and depth of data collected will also increase. Several projects have utilized aspects of these datasets [13–16], and more are in preparation. The potential scope of application for these data is broad and is anticipated to impact the field simply by being the first public dataset of its scale that has been collected and reported in a crop sciences using standardized protocols and formats, thus defining standards for data collection, formatting, and access for maize and other species.

## Limitations

These datasets contain missing data. In the phenotypic and genotypic datasets, missing data is left blank instead of indicated by ‘null’ or zero to not interfere with software compatibility and interpretation. The only exception is for traits extracted from 2014 and 2015 ear imaging data, which are demarcated with ‘NA’.

For weather datasets, raw files reported by sensors are not provided because machine data were calibrated based on information from nearby weather stations to ensure accuracy (e.g., if the wind vane was set improperly, a calibration correction was required). Instead, only the cleaned version of the file is reported to reduce misinterpretation.

The geographic locations of field locations are not identical across years due to crop rotation management practices. Along with the field location code, the GPS coordinates are reported. While the germplasm used in the experiments is publicly accessible, it was not generated directly by national public genebanks. Seed access and availability are handled by the G2F collaborators directly.

## Data Availability

The data described in this Data Note can be freely and openly accessed at CyVerse via the following Digital Object Identifiers (DOIs): https://www.doi.org/10.25739/frmv-wj25, https://www.doi.org/10.25739/9wjm-eq41, https://www.doi.org/10.25739/kjsn-dz84, https://www.doi.org/10.25739/yjnh-kt21, https://www.doi.org/10.25739/w560-2114 and 10.7946/P2C34P. See Table 1 and reference list for details and links to the data.

## Abbreviations

G2F: Genomes to fields
G × E: Genotype-by-environment
GBS: Genotyping-by-sequencing
RGB: Red/green/blue
DOI: Digital Object Identifier
